# Surveillance of Zika virus infection in the EU/EEA, June 2015 to January 2017

**DOI:** 10.2807/1560-7917.ES.2017.22.41.17-00254

**Published:** 2017-10-12

**Authors:** G Spiteri, B Sudre, A Septfons, J Beauté, on behalf of the European Zika surveillance Network

**Affiliations:** 1 European Centre for Disease Prevention and Control (ECDC), Stockholm, Sweden; 2 Santé publique France, Paris, France; 3 European Programme for Intervention Epidemiology Training (EPIET), European Centre for Disease Prevention and Control (ECDC), Stockholm, Sweden; 4 The members of the European Zika surveillance Network are listed at the end of the article

**Keywords:** Zika, vector-borne infections, surveillance, emerging diseases, re-emerging diseases

## Abstract

Surveillance of Zika virus (ZIKV) infection in the European Union/European Economic Area (EU/EEA) was implemented in 2016 in response to the large outbreak reported in the Americas in 2015 associated with an increased number of infants born with microcephaly. Between June 2015 and January 2017, 21 EU/EEA countries reported 2,133 confirmed cases of ZIKV infection, of whom 106 were pregnant women. Cases infected in the Caribbean constituted 71% of reported cases. Almost all cases (99%) were most probably infected by mosquito bite during travel outside continental Europe, while only 1% were transmitted sexually. Considering that 584 imported cases were reported between May and October 2016 among residents of areas with established presence of *Aedes albopictus*, the absence of autochthonous vector-borne cases suggests that *Ae. albopictus* is not an efficient vector for ZIKV infection.

## Introduction

Zika virus (ZIKV) was first identified in humans in the 1950s. The first large outbreaks, however, were not reported until 2007 from the Island of Yap (Micronesia) in 2007 [1] and from French Polynesia in 2013–14 [2]. In 2015, an outbreak of unprecedented magnitude was reported in the Americas temporally associated with an increased number of infants born with microcephaly [3]. On 1 February 2016, the World Health Organization (WHO) declared that “*the recent cluster of microcephaly cases and other neurological disorders reported in Brazil, following a similar cluster in French Polynesia in 2014, constitutes a Public Health Emergency of International Concern*” and encouraged the investigation of an association with ZIKV which at the time had not been confirmed [4]. 

In March 2016, the European Union (EU) Health Security Committee approved an interim case definition for surveillance of ZIKV infection [5] and the European Centre for Disease Prevention and Control (ECDC) proceeded to develop surveillance at the level of the European Union/European Economic Area (EU/EEA). The objectives were the early detection of locally acquired cases and timely reporting of travel-associated cases, particularly those residing in areas in the EU/EEA where *Aedes albopictus* or *Ae. aegypti* are established (receptive areas), to trigger appropriate control measures. We here report the results of ZIKV infection surveillance among EU/EEA residents in the period from 2015 to 2017. Since *Ae. aegypti* is only established on Madeira in the EU/EEA, it was not considered for this analysis.

## Methods

Epidemiological surveillance of ZIKV infection in the EU/EEA was implemented in 2016 and is carried out by nominated representatives from EU/EEA countries, the European Zika surveillance network, under the coordination of ECDC. ECDC has published interim guidance outlining the investigation and testing of suspected cases [6], however, some countries have implemented their own criteria for testing and reporting over time. Surveillance is based on weekly reporting to ECDC of case-based or aggregated data on confirmed cases. The option of reporting aggregated data aims to reduce the reporting burden on the countries, particularly in case of large local outbreaks in Europe. Confirmed cases are defined based on (i) detection of ZIKV nucleic acid, detection of ZIKV antigen or isolation of ZIKV from a clinical specimen, (ii) detection of ZIKV-specific IgM antibodies in a serum sample and confirmation by neutralisation test or (iii) seroconversion or fourfold increase in the titre of ZIKV-specific antibodies in paired serum samples [5]. Case-based data include information on age, sex, date of onset, date of notification, importation status, probable place of infection, place of residence, place of notification, pregnancy status and probable mode of transmission. Aggregated data include the number of cases per week by pregnancy status and residence in receptive or non-receptive areas for imported cases and the number of cases per week by probable place of infection, pregnancy status and mode of transmission for locally acquired cases. Cases are reported only if diagnosed in continental Europe (which we also take to include Cyprus, Iceland, Ireland, Malta and the United Kingdom (UK)) or in selected outermost regions (Azores, Canary Islands, Madeira) [7] of the European Union. Data collection started in June 2016 and is ongoing, but EU/EEA countries also reported retrospectively cases of ZIKV infection that had occurred from 2015 onwards. 

For the present analysis, we extracted data from the ZIKV surveillance database on 14 March 2017. The analysis included description of reported cases over time, by importation status, age, sex and pregnancy. Areas where *Ae. albopictus* was established were defined based on data published by the VectorNet project, a joint initiative of the European Food Safety Authority and ECDC that supports the collection of data on vectors and pathogens in vectors, related to both animal and human health [8]. 

The k-sample median test was used to compare medians using STATA, version 14 (StataCorp, College Station, United States (US)).

## Results

### Overview

**Figure 1:**
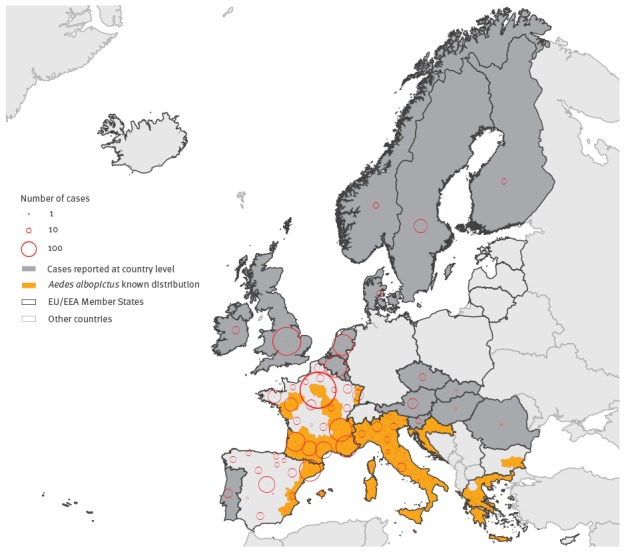
Number of cases of Zika virus infection by place of residence (NUTS2) and established presence of *Aedes albopictus* as at 14 March 2017, 21 EU/EEA countries^a^, week 26/2015–week 5/2017 (n = 1,881)

Until 13 March 2017, 21 EU/EEA countries (total population: 375 million) reported 2,133 confirmed cases of ZIKV infection to ECDC, with reporting dates between week 26, 2015 (the week starting on 29 June 2015) and week 5, 2017 (the week ending on 5 February 2017). These included 2,090 imported cases, 21 locally acquired non-vector borne cases and 22 cases with importation status reported as unknown. France reported the largest number of cases (1,141 cases) followed by Spain (306 cases), the UK (199 cases) and Belgium (128 cases). Overall, of the 1,881 cases with known region of residence, 815 (43%) lived in areas where *Ae. albopictus* was established (Figure 1).

EU/EEA: European Union/European Economic Area.

^a^ Austria, Belgium, Czech Republic, Denmark, Finland, France, Greece, Hungary, Ireland, Italy, Luxembourg, Malta, the Netherlands, Norway, Portugal, Romania, Slovakia, Slovenia, Spain, Sweden and the United Kingdom.

Cases reported from countries where *Aedes albopictus* is not established in the continental part of the country are displayed at country level.

### Place of infection

The place of infection was reported for 1,819 (87%) of the imported cases. The largest proportion was reported to have been infected in the Caribbean (71%). Infections were also acquired in South America (17%) and Central America (11%), and much smaller proportions in Asia, Africa, Oceania and North America (all < 1%). The most frequently reported places of infection were Guadeloupe (489 cases), Martinique (421 cases) and the Dominican Republic (146 cases). Table 1 shows the 10 most common countries and overseas territories from where cases were imported. The three highest ranked places of infection varied by reporting country. Among the three European countries reporting most cases, France reported most cases imported from Guadeloupe, Martinique and French Guiana, Spain from the Dominican Republic, Colombia and Venezuela and the UK from Barbados, Jamaica and Saint Lucia (data not shown).

**Table 1:** Most commonly reported destination countries and overseas territories in imported cases of Zika virus infection, 21 EU/EEA countries, week 26/2015 to week 5/2017 (n = 2,090)

Rank	Destination country	Number	%
1	Guadeloupe	489	26.9
2	Martinique	421	23.1
3	Dominican Republic	146	8.0
4	Colombia	83	4.6
5	Mexico	81	4.5
6	Brazil	68	3.7
7	Barbados	53	2.9
8	Venezuela	52	2.9
9	Nicaragua	51	2.8
10	Suriname	48	2.6
Other		327	18.0
Total documented		1,819	100
Not documented		271	NA
Total		2,090	NA

EU/EEA: European Union/European Economic Area. NA: not applicable.

Data as at 14 March 2017. Percentages are based on the cases with documented destination. Excludes cases reported with unknown importation status (Belgium: 19 cases, France: three cases), cases with reported sexual transmission (20 cases) and one mother-to-child transmission in the EU/EEA. The total number of cases with documented destination includes eight cases where the destination was reported as ‘French overseas department’.

### Time of infection

**Figure 2:**
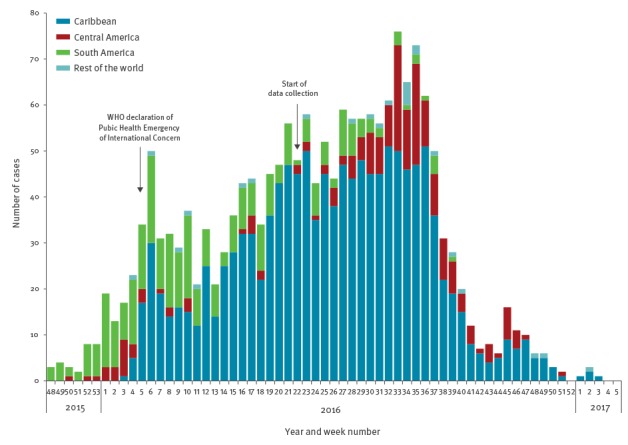
Number of cases of Zika virus infection by week of reporting and probable region of infection, 21 EU/EEA countries^a^, week 48/2015–week 5/2017 (n = 1,811)

The first imported case was reported in week 26, 2015. The weekly number of imported cases started to increase during the last weeks of 2015, peaking during week 33, 2016 when 85 imported cases were reported in one week. Other intermediate peaks were observed in weeks 6 and 23, 2016 (Figure 2). Cases then declined rapidly from week 35, 2016 onwards, although there was a slight increase in cases around week 45, 2016. Cases reported as infected in the Caribbean peaked during week 6 and weeks 21–36, 2016, cases infected in South America between weeks 1 and 10, 2016, and cases infected in Central America between weeks 33 and 35, 2016. The date of onset was reported for 1,608 cases (75%). The median lag between date of onset and date of notification was one week and ranged between 0 and 14 weeks.

EU/EEA: European Union/European Economic Area.

^a^ Austria, Belgium, Czech Republic, Denmark, Finland, France, Greece, Hungary, Ireland, Italy, Luxembourg, Malta, the Netherlands, Norway, Portugal, Romania, Slovakia, Slovenia, Spain, Sweden and the United Kingdom.

Excludes eight cases where the destination was reported as ‘French overseas department’.

### Demographics

Of the 2,133 cases, 1,048 were female, 696 were male, and for 389 (18%) sex was not reported (Table 2).

**Table 2:** Main characteristics of the cases of Zika virus infection, 21 EU/EEA countries^a^, week 26/2015–week 5/2017 (n = 2,133)

Characteristic	Number	%
**Sex **		
Female	1,048	60.1
Male	696	39.9
Not documented	389	
**Age group (years) **		
0–4	13	0.8
5–14	51	2.9
15–24	138	7.9
25–34	499	28.7
35–44	401	23.1
45–54	282	16.2
55–64	236	13.6
≥ 65	118	6.8
Not documented	395	
**Region visited **		
Africa	4	0.2
Asia	15	0.8
Caribbean	1,278	70.6
Central America	205	11.3
Oceania	3	0.2
North America	3	0.2
South America	303	16.7
No travel	21	
Not documented	301	
**Mode of transmission **		
Mosquito	1,715	98.7
Mother-to-child	2	0.1
Sexual	20	1.2
Not documented	396	
**Pregnancy status** ^b^		
Pregnant	105	16.0
Not pregnant	551	84.0
Not documented	52	

EU/EEA: European Union/European Economic Area; NA: not applicable.

^a^ Austria, Belgium, Czech Republic, Denmark, Finland, France, Greece, Hungary, Ireland, Italy, Luxembourg, Malta, the Netherlands, Norway, Portugal, Romania, Slovakia, Slovenia, Spain, Sweden and the United Kingdom.

^b^ Includes only female individuals aged 15–49 years (n = 708). In addition, one pregnant woman was reported with unknown age.

Data as at 14 March 2017. Percentage is calculated over documented values, and for region visited, among cases travelling abroad.

Among those for whom sex was reported, 60% were female. Among all female cases, pregnancy status was reported for 92%; 106 ZIKV-infected women were reported to be pregnant. Age was reported for 1,738 cases (81%). Of these, four were aged one month or younger, while the largest proportion was 25–34 (29%) and 35–44 years-old (23%). The median age was 38 years and did not vary by sex (p = 0.2, k-sample test). Pregnant women were younger (median age: 31 years) when compared with non-pregnant women (median age: 39 years, p < 0.01, k-sample test). The overall male-to-female ratio was 0.7; it was lowest among 15–24 year-olds (0.4).

### Mode of transmission

Nearly 99% of cases with reported mode of transmission were infected by mosquito bite during travel outside continental Europe. Among the imported cases, one case of mother-to-child transmission was reported associated with maternal travel to Brazil. There were no locally acquired vector-borne ZIKV infections among reporting countries during the period under surveillance. Sexual transmission was reported for 20 cases, all locally acquired, from six countries (France: 12 cases, Italy: two cases, the Netherlands: two cases, Portugal: one case, Spain: two cases and the UK: one case). Of the 20 cases where sex was the reported mode of transmission, 19 were women. Their ages ranged from 17 to 63 years (median: 33 years). The age and sex of one case were not reported. Three of the women infected through sexual transmission were pregnant. One locally acquired case of mother-to-child transmission was reported from Spain.

## Discussion

The demographic data of cases reported in this study were very similar to those reported among US travellers (60% women) [9]: considering the risk of severe pregnancy outcomes [10], it is expected that women of reproductive age, and particularly pregnant women, are more frequently tested. In our study, approximately 16% of infected women of reproductive age were pregnant, which is suggestive of increased testing in pregnant women. Indeed, the male-to-female sex ratio for persons of reproductive age (15–49 years) was 0.7. Other possible reasons for this pattern could reflect health-seeking behaviour among women, particularly those of child-bearing age, differences in exposure to mosquito bites and possibly sexual transmission, which has been reported more often from men to women than from women to men [11]. The proportion of sexually transmitted cases was approximately 1%, similar to what has been reported from the US, and in line with suggestions of limited potential for sexual transmission [12,13].

A study from France reported that around 30% of imported cases were residents in areas where *Ae. albopictus* was established during the vector activity period [14]. Our data indicated an even higher proportion (43%): in addition to France, all Greek, Italian, Maltese and Slovenian travellers, and almost half of the Spanish travellers (45%) resided in such areas. The absence of local transmission despite the large number of cases among travellers (many of whom may have been viraemic) returning to these areas between the beginning of May and the end of October (584 cases among returning travellers during that time period) suggests that *Ae. albopictus* is probably not an efficient vector for ZIKV, as reported in other studies [15,16]. Nevertheless, surveillance should continue as *Ae. albopictus* has been implicated in the 2007 ZIKV outbreak in Gabon [17] and possibly in Mexico [18]. European mosquito populations have shown some competence for ZIKV particularly at higher temperatures under laboratory conditions [19].

The trends in reported cases reflected the progression of the epidemic in the Americas, starting in South America in early 2016 and then progressing to the Caribbean and eventually to Central America in late 2016. Most cases were associated with travel to the Caribbean which may be explained by the travel pattern of European residents, the stage of the epidemics at the time of major holiday periods and the higher intensity of the epidemics in insular settings [20]. Using a mathematical model developed for dengue importation, a study estimated between 116 and 355 symptomatic ZIKV infections imported to Europe by travellers from Brazil in 2016 [21]. Our surveillance data covered approximately 60% of the EU/EEA population, and 68 ZIKV infections with a probable origin in Brazil were reported, which, when extrapolated to the whole EU/EEA population, is consistent with the lower limits of the prediction.

Interestingly, our data show that surveillance in the EU/EEA was able to capture the cases returning from Africa, Asia and Oceania. Surveillance of the cases imported to the EU/EEA could therefore serve as an indicator (although probably not a very sensitive one) of emerging and ongoing transmission, particularly in countries with limited testing capacity, and it could contribute to the evidence base used for the WHO ZIKV country classifications.

European surveillance data may underestimate the importation of ZIKV cases in Europe. It is likely that most cases were tested after developing symptoms and asymptomatic ZIKV cases, particularly in non-pregnant women, are therefore likely to be under-represented. It is also likely that cases are underestimated in areas and countries without established *Ae. albopictus* populations as well as, in countries with established populations, at times when *Ae. albopictus* is not active. In addition, some groups may be tested less frequently, e.g. travellers returning from countries without documented transmission. Other groups may be more likely to be tested, e.g. pregnant women. Laboratory capacities vary across countries and access to testing can therefore not be expected to be uniform across Europe. Data on the diagnostic method used for diagnosis were not available and we can therefore not know what proportion of cases were diagnosed through nucleic acid amplification testing, isolation or serology. It is likely that most of the diagnoses during the period under surveillance were made through nucleic acid amplification testing; however, the impact of serology and cross-reactions with other circulating arboviruses such as dengue virus and chikungunya virus cannot be to assessed. Finally, further testing and validation of cases might mean that some cases have been reclassified or excluded since the extraction of the data on 14 March 2017. 

## Conclusion

ECDC rapidly implemented surveillance of ZIKV infection following the Health Security Committee decision, with two-thirds of EU/EEA countries reporting their cases every week. These data were used to produce a ZIKV infection surveillance atlas, which was updated each week. In addition, key results were discussed on a weekly basis at ECDC and presented in the Communicable Disease Threat Report [22].

Prevention of ZIKV in Europe is challenging as the vast majority of cases are imported and, apart from travel health clinics, there are limited opportunities to provide targeted prevention advice. Efforts need to be made to strengthen travel health advice before peak travel periods, targeting particularly pregnant women and their partners. Surveillance of ZIKV at the European level has proven to be beneficial during a rapidly evolving global public health emergency, with active participation of the majority of EU/EEA countries. Further development of the system will aim to capture pregnancy outcome to provide understanding of the impact of ZIKV in Europe. The European ZIKV surveillance system could serve as a model for future emerging infections.

## Notes

**Members of the European Zika surveillance Network:** Stephan W. Aberle, Medical University of Vienna, Center for Virology, Vienna, Austria. Marjan Van Esbroeck, National Reference Center for Arboviruses, Antwerp, Belgium. Helena Šebestová, National Institute of Public Health, Czech Republic. Anders Koch, Statens Serum Institut, Copenhagen, Denmark. Jussi Sane, Infectious Disease Control Unit, Department of Health Security, National Institute for Health and Welfare, Helsinki, Finland. Stella Laporal, Santé publique France, Paris, France. Danai Pervanidou, Hellenic Center for Disease Control and Prevention, Athens, Greece. Bognár Zsófia, National Center for Epidemiology, Budapest, Hungary Sarah Jackson, Health Protection Surveillance Centre, Dublin, Ireland. Caterina Rizzo, Istituto Superiore di Sanità, Rome, Italy. Tanya Melillo, Infectious Disease Prevention and Control Unit, Msida, Malta. Janneke W. Duijster, National Institute for Public Health and the Environment (RIVM), Bilthoven, Netherlands. Bernardo R. Guzmán-Herrador, Department of Infectious Disease Epidemiology, Norwegian Institute of Public Health, Oslo, Norway. Marina Ramos, Directorate-General of Health, Lisbon, Portugal. Ionel Iosif, National Institute of Public Health, Bucharest, Romania. Maja Sočan, National Institute of Public Health, Ljubljana, Slovenia. Beatriz Fernandez-Martinez, Spanish National Centre of Epidemiology, Madrid, Spain. Elsie Ydring, Public Health Agency of Sweden, Stockholm, Sweden. Joanne Freedman, Public Health England, London, United Kingdom.
